# ‘Just part of the job’ – understanding work‐related injuries and safety culture in companion animal veterinary practices

**DOI:** 10.1111/jsap.70039

**Published:** 2025-11-12

**Authors:** J. S. P. Tulloch, I. Schofield, R. Jackson, M. Whiting

**Affiliations:** ^1^ Institute of Infection, Veterinary and Ecological Sciences University of Liverpool Liverpool UK; ^2^ CVS UK Ltd Diss UK

## Abstract

**Objectives:**

To examine the prevalence and types of work‐related injuries in companion animal practices, explore the context of their occurrence and the behaviours of injured persons.

**Methods:**

A mixed‐methods analysis of a cross‐sectional online survey of UK employees of a consolidated group of veterinary practices.

**Results:**

Of 647 respondents, 77.6% experienced a work‐related injury during their career. In the previous year, 60.2% of veterinary nurses and 58.3% of veterinarians were injured, most frequently in consultation rooms, prep areas, kennels and reception. Animal‐related injuries were the most prevalent injury type. Injuries frequently occurred during cat restraint, anaesthetic recovery and clinical examinations. Needlestick injuries made up 15.8% of veterinary injuries. 16.3% of injured nurses and 19.4% of injured vets attended hospital. 34.3% of nurses and 25.1% of vets needed more than a week to recover from their injuries. Fewer than 10% took time off work, often due to a sense of duty, the ability to manage a reduced workload or simply wanting to “get on with it”. Most injuries to vets went unreported, due to perceived time pressures or the belief that the injury was minor. Around half adjusted their behaviour post‐injury, becoming more cautious or changing handling techniques.

**Clinical significance:**

This study reveals a high rate of work‐related injuries in companion animal practices. A poor injury culture, marked by presenteeism, often downplaying risks, and hindering safety. To reduce injuries, a shift towards shared responsibility and reflective learning is needed, driven by strong leadership and open communication.

## INTRODUCTION

The veterinary industry has some of the highest levels of work‐related injuries (US Bureau of Labor Statistics, 2023). In the United Kingdom (UK), around two‐thirds of veterinarians are injured annually (Mills, 2015). It has been estimated that 95% of companion animal veterinarians have been injured within the last 5 years (Epp & Waldner, 2012), with between 26% and 34% being injured in the last 12 months (Fritschi et al., 2006; Johnson & Fritschi, 2024).

Companion animal veterinary practices present various workplace injury risks, primarily due to animals, handling of sharp instruments and exposure to hazardous substances (Epp & Waldner, 2012). Animal‐related injuries (cat bites and scratches, and dog bites) are the most prevalent cause of injury (Johnson & Fritschi, 2024; Tulloch et al., 2023; Voss et al., 2023), often occurring during the restraint, clinical examination or administration of drugs to said animal (Tulloch et al., 2023). Many of these animal‐related injuries will require medical attention, with varied estimates of the number requiring hospital treatment (4% to 16%) (Lucas et al., 2009; Tulloch et al., 2023). Musculoskeletal injuries can occur from lifting heavy animals or maintaining awkward postures during procedures, with 46% to 49% of veterinary surgeons receiving one annually (Epp & Waldner, 2012; Fritschi et al., 2006). Needlestick injuries and exposure to anaesthetic gases, disinfectants or medications pose additional health risks (Epp & Waldner, 2012; Johnson & Johnstone, 2025). It has been estimated that 57% to 65% of veterinarians receive a needlestick annually (Epp & Waldner, 2012; Johnson & Fritschi, 2024).

Despite there being literature describing injuries to veterinarians, there is little describing the context of those injuries, their consequences, nor the health seeking or reporting behaviours of the injured person (IP). There is also scant information about other roles being injured in a companion animal veterinary practice (i.e. veterinary nurses, receptionists, animal care assistants). A strong safety culture within a workplace, defined by shared values, attitudes and behaviours regarding workplace safety, plays a crucial role in mitigating risks and preventing injuries (European Agency for Safety and Health at Work, 2023). It appears that there is some shared culture between veterinary roles, as they have a consistent definition of a work‐related injury being one causing “physical trauma with pain”; however, one in ten also incorrectly believe that preceding events must be an accident for something to be defined as an injury (Furtado et al., 2024). In UK companion animal practices, injury prevalence, health seeking and reporting behaviours remain underexplored. Understanding the relationship between safety culture and work‐related injuries is essential for developing targeted interventions, improving training programs and fostering a safer working environment for veterinary professionals.

This paper aims to examine the prevalence and types of work‐related injuries in companion animal practices, explore the context of their occurrence and the behaviours of injured persons. The study seeks to provide actionable insights for enhancing occupational health in veterinary settings.

## MATERIALS AND METHODS

An online cross‐sectional survey was designed and distributed to all UK employees of CVS Group plc, working in companion animal clinical practices. CVS Group Ltd is a provider of veterinary services in the UK and Australia. They have over 450 companion animal first opinion veterinary practices and nine referral hospitals in the UK and represent a large share of the UK companion animal veterinary sector. The survey was distributed through weekly emails and was open for 3 months between December 6, 2022, and March 6, 2023. The survey was piloted with 20 CVS employees. To enhance recruitment, an incentive of a year’s supply of tea, coffee and biscuits for the practices of five randomly selected respondents was provided.

Respondents were asked about personal demographics (i.e. sex, age, nationality) and details of their job role, number of hours worked and whether they perform clinical work. Then about whether they had acquired a work‐related injury during their career in the veterinary sector. If they had, they were presented with a series of questions about their most recent injury and their respondent‐defined most severe injury. These included questions about injury context, medical consequences, resultant behaviour change, time off work and reporting to their employer. If an animal was involved in the circumstances leading up to the injury, this was classified as an animal‐related injury.

Demographic characteristics of respondents were described. Overall injury prevalence was calculated and stratified by job role. Logistic regression was performed to identify any association between demographic variables and ever receiving an injury. Variables taken forward for multivariable analysis were selected through substantive knowledge and statistical significance (i.e. where P ≤ 0.3). Hosmer–Lemeshow tests were performed to assess goodness of fit, and analysis of a correlation matrix of independent variables was used to assess collinearity.

The data were subsequently stratified from the remainder of the analysis into the following groups: administrative, receptionists, animal care assistants (ACA), veterinary nurses (VN) and veterinary surgeons (VS). The types of injury acquired were described, and it was recorded whether they were reportable to the Health and Safety Executive (HSE). In the UK, under the Reporting of Injuries, Diseases and Dangerous Occurrences Regulation 2013 (RIDDOR), an employer legally must report work‐related accidents that result in specific injuries to the HSE (Health & Safety Executive, 2013). These injuries include any that leave them incapacitated for more than 7 days, fractures other than to digits, loss of consciousness, among others. We performed more focused analysis on animal‐related injuries and the procedures occurring at the point of injury, as we anticipated that these would represent the majority of injuries to clinical staff.

The analysis of questions relating to the context of injury, attitude of injured persons and reporting culture was carried out descriptively. Open text questions were analysed using an iterative thematic analysis (Vears & Gillam, 2022). Initially, the responses were read through with the researchers making note of any initial impressions; secondly, the researchers carried out initial “coding” by reading items individually and labelling important concepts. As more text was incorporated into the analysis, codes were refined, combined or renamed to more accurately represent the information conveyed by respondents. Eventually, codes could be grouped into overall categories or “themes”.

Data masking was used to protect personally identifiable information of respondents; this was performed through aggregation of data or redacting of quotes. The survey instrument is available upon reasonable request to the corresponding author.

The study received ethical approval from the University of Liverpool Veterinary Research Ethics Committee (VREC1256).

## RESULTS

Six hundred and forty‐seven individuals, out of a possible 6163, consented to take part in the survey. This represented a response rate of 10.5%. VS respondents were representative of the UK profession in terms of age (median national age 30 to 39) and ethnicity (3.5% ethnic minority groups nationally) (Table 1) (Robinson, Edwards, Mason, et al., 2019). However, female respondents were overrepresented (59% of veterinarians are female nationally). VNs were broadly representative in terms of sex (96.8% female nationally), age (median age of 30 to 39 nationally) and ethnicity (1.9% ethnic minority groups nationally) (Robinson, Edwards, Akehurst, et al., 2019). National demographics of companion animal ACAs, administrators and receptionists are currently not available.

**Table 1:** Demographics of respondents to a survey on veterinary work‐related injuries in companion animal practices

	Administrative staff (*n* = 91) (95% CI)	Reception staff (*n* = 100) (95% CI)	Animal care assistants (*n* = 61) (95% CI)	Veterinary nurses (*n* = 208) (95% CI)	Veterinary surgeons (*n* = 187) (95% CI)
**Sex (female)**	91.2% (84.0–95.8)	99.0% (94.6–100)	86.9% (75.8–94.2)	95.1% (91.3–97.7)	83.9% (77.8–88.9)
**Age**					
<20	0	2.0% (0.2 to 7.0)	1.6% (0.0 to 8.8)	0	0
20 to 29	5.5% (1.8 to 12.4)	32.0% (23.0 to 42.1)	54.1% (40.9 to 66.9)	28.8% (22.8 to 35.5)	23.0% (17.2 to 29.7)
30 to 39	20.9% (13.1 to 30.7)	15.0% (8.6 to 23.5)	26.2% (15.8 to 39.1)	35.0% (28.6 to 42.0)	36.9% (30.0 to 44.3)
40 to 49	45.1% (34.6 to 55.8)	11.0% (5.6 to 18.8)	6.6% (1.8 to 16.0)	28.8% (22.8 to 35.5)	25.7% (19.6 to 32.6)
50 to 59	24.2% (15.8 to 34.3)	22.0% (14.3 to 31.4)	8.2% (2.7 to 18.1)	5.3% (2.7 to 9.3)	9.6% (5.8 to 14.8)
60+	4.4% (1.2 to 10.9)	18.0% (11.0 to 27.0)	3.3% (0.4 to 11.4)	1.9% (0.5 to 4.9)	4.8% (2.2 to 8.9)
**Ethnicity**					
White	97.8% (92.1 to 99.7)	98.0% (93.0 to 99.8)	98.4% (91.2 to 100)	97.1% (93.8 to 98.9)	95.2% (91.0 to 97.8)
All other ethnic groups combined	2.2% (0.3 to 7.9)	2.0% (0.2 to 7.0)	1.6% (0.0 to 8.8)	2.9% (1.1 to 6.2)	4.8% (2.2 to 9.0)
**Hours of employment**					
<10 hours	0	0	0	0.5% (0.0 to 2.7)	1.6% (0.3 to 4.6)
11 to 20 hours	4.4% (1.2 to 10.9)	8.0% (3.5 to 15.2)	3.3% (0.4 to 11.4)	9.1% (5.6 to 13.9)	7.5% (4.2 to 12.2)
21 to 30 hours	12.1% (6.2 to 20.6)	33.0% (23.9 to 43.1)	16.4% (8.2 to 28.1)	13.9% (9.5 to 19.4)	13.9% (9.3 to 19.7)
31 to 40 hours	80.2% (70.6 to 87.8)	54.0% (43.7 to 64.0)	72.1% (59.2 to 82.9)	71.1% (64.5 to 77.2)	66.3% (59.1 to 73.0)
41 to 50 hours	3.3% (0.7 to 9.3)	5.0% (1.6 to 11.3)	8.2% (2.7 to 18.1)	5.3% (2.7 to 9.3)	10.2% (6.2 to 15.4)
50 + hours	0	0	0	0	0.5% (0.0 to 2.9)
**Out of hours**					
No out of hours	67.0% (56.4 to 76.5)	51.0% (40.8 to 61.1)	31.1% (19.9 to 44.3)	38.5% (31.8 to 45.4)	25.7% (19.6 to 32.6)
<10 hours	28.6% (19.6 to 39.0)	48.0% (37.9 to 58.2)	59.0% (45.7 to 71.5)	53.4% (46.3 to 60.3)	65.8% (58.5 to 72.5)
11 to 20 hours	3.3% (0.7 to 9.3)	0	4.9% (1.0 to 13.7)	2.4% (0.8 to 5.5)	3.2% (1.2 to 6.9)
21 to 30 hours	0	1.0% (0.0 to 5.4)	3.3% (0.4 to 11.4)	2.9% (1.1 to 6.2)	3.2% (1.2 to 6.9)
31 to 40 hours	0	0	1.6% (0.0 to 8.8)	1.0% (0.1 to 3.4)	1.6% (0.3 to 4.6)
41 to 50 hours	1.1% (0.0 to 6.0)	0	0	1.0% (0.1 to 3.4)	0
50 + hours	0	0	0	1.0% (0.1 to 3.4)	0.5% (0.0 to 2.9)
**Clinical responsibilities**					
No clinical responsibilities	80.2% (70.6 to 87.8)	94.0% (87.4 to 97.8)	18.0% (9.4 to 30.0)	2.4% (0.8 to 5.5)	0
<10 hours	16.5% (9.5 to 25.7)	5.0% (1.6 to 11.3)	8.2% (2.7 to 18.1)	9.1% (5.6 to 13.9)	5.3% (2.6 to 9.6)
11 to 20 hours	3.3% (0.7 to 9.3)	0	13.1% (5.8 to 24.2)	17.3% (12.4 to 23.2)	15.5% (10.6 to 21.5)
21 to 30 hours	0	0	16.4% (8.2 to 28.1)	27.9% (21.9 to 34.5)	23.0% (17.2 to 29.7)
31 to 40 hours	0	1.0% (0.0 to 5.4)	42.6% (30.0 to 56.0)	41.3% (34.6 to 48.4)	47.1% (39.7 to 54.5)
41 to 50 hours	0	0	1.6% (0.0 to 8.8)	1.4% (0.3 to 4.2)	8.6% (5.0 to 13.5)
50 + hours	0	0	0	0.5% (0.0 to 2.7)	0.5% (0.0 to 2.9)

**FIG 1:**
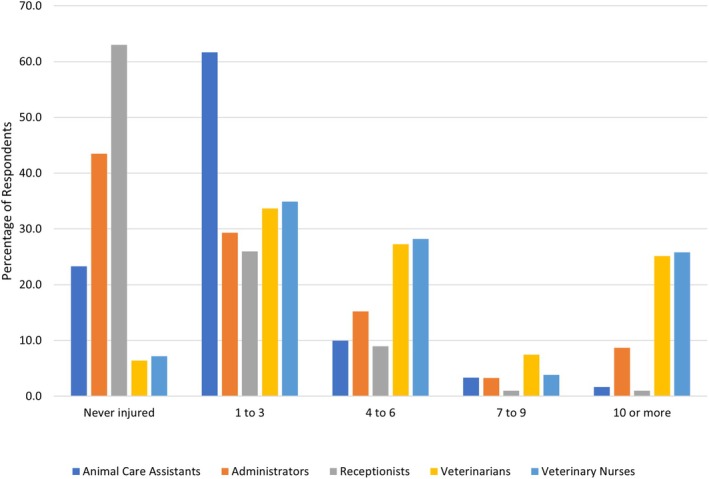
Frequency of work‐related injuries over a career in companion animal practices

More than three‐quarters of all staff had acquired a work‐related injury in the veterinary industry during their career (77.6%). This was highest for VS (93.6%), followed by VNs (92.8%), ACAs (75.4%), administrators (56.0%) and receptionists (37.0%). Veterinarians and VNs reported the highest frequency of injuries (Fig. 1).

Univariable analysis to identify which demographic variables were associated with a respondent having ever acquired a veterinary work‐related injury identified the following significant variables: sex, role, hours worked, out of hours worked and clinical hours worked (Table 2). When taking forward variables for multivariable analysis, “clinical hours” was removed as there was strong collinearity with “role”. The multivariable analysis showed that role and hours worked were the variables with significance. All roles had higher odds of having acquired an injury, when adjusted for sex, hours worked and OOH worked, than receptionists. The roles with the highest odds of injury were VSs and VNs. Only those that worked between 21 and 40 hours a week had higher odds of injury compared to those working less than 21 hours; those working more than 40 hours had similar injury odds to those working the fewest hours.

**Table 2:** Univariable and multivariable logistic regression exploring demographic factors associated with acquiring a veterinary work‐related injury in companion animal practices

	Univariable analysis		Multivariable analysis	
	Odds ratio (95% CI)	*P*‐value	Odds ratio (95% CI)	*P*‐value
**Sex** (ref: female)	2.2 (1.0 to 5.4)	.06	1.2 (0.5 to 3.3)	.63
**Age** (ref: ≤29 years old)				
30 to 39	1.4 (0.9 to 2.3)	.19		
40 to 49	1.3 (0.8 to 2.2)	.34		
50 to 59	0.8 (0.5 to 1.5)	.53		
60+	0.8 (0.4 to 1.7)	.50		
**Ethnicity** (ref: white)				
All other ethnic groups combined	2.7 (0.8 to 17.0)	.19		
**Role** (ref: receptionist)				
Animal care assistant	5.2 (2.6 to 10.9)	<.001	4.9 (2.4 to 10.5)	<.001
Administrator	2.2 (1.2 to 3.9)	<.01	2.0 (1.1 to 3.7)	.03
Veterinary nurse	21.9 (11.6 to 42.9)	<.001	24.1 (12.2 to 50.4)	<.001
Veterinary surgeon	24.8 (12.6 to 52.7)	<.001	28.5 (13.6 to 64.2)	<.001
**Weekly hours worked** (ref: = <20 hours)				
21 to 30	1.4 (0.7 to 2.9)	.32	4.1 (1.6 to 10.4)	<.01
31 to 40	2.3 (1.2 to 4.2)	.01	4.4 (1.9 to 9.9)	<.001
41+	1.6 (0.7 to 4.1)	.28	1.9 (0.6 to 5.8)	.28
**Weekly “out of hours” worked** (ref: 0 hours)				
>0 to 10	1.7 (1.2 to 2.5)	<.01	0.96 (0.6 to 1.6)	.87
11 to 20	3.1 (0.8 to 19.7)	.14	1.9 (0.4 to 13.2)	.45
≥21	5.1 (1.5 to 32.2)	.03	1.9 (0.5 to 13.0)	.44
**Weekly clinical hours worked** (ref: 0 hours)				
>0 to 10	10.3 (4.5 to 27.9)	<.001		
11 to 20	6.9 (3.6 to 14.2)	<.001		
21 to 30	27.3 (11.7 to 80.0)	<.001		
31 to 40	16.0 (9.0 to 30.2)	<.001		
41+	5.8 (2.1 to 20.7)	<.01		
**Knowledge of Health and Safety Protocol** (ref: no knowledge)	1.38 (0.8 to 2.2)	.19		

The last 12 months’ annual work‐related injury prevalence for administrators was 12.1% (95% CI 6.2 to 20.6), for receptionists 21.0% (13.5 to 30.3), for ACAs 62.3% (49.0 to 74.4), for VNs 60.6% (53.4 to 67.3) and for VS 58.3% (50.9 to 65.4). Over 90% of all staff were injured within the practice. The most prevalent areas for injuries were the consult room, the prep room, the ward/kennels area and reception (Tables 3 and S1).

**Table 3:** Context of recent veterinary work‐related injuries in companion animal practices

	Administrative staff (*n* = 51) (95% CI)	Reception staff (*n* = 36) (95% CI)	Animal care assistants (*n* = 45) (95% CI)	Veterinary nurses (*n* = 192) (95% CI)	Veterinary surgeons (*n* = 175) (95% CI)
**When did the injury occur?**					
<3 months ago	7.8% (2.2 to 18.9)	13.9% (4.7 to 29.5)	40.0% (25.7 to 55.7)	28.1% (21.9 to 35.1)	29.7% (23.1 to 37.1)
3 to 6 months ago	5.9% (1.2 to 16.2)	16.7% (6.4 to 32.8)	22.2% (11.2 to 37.1)	16.7% (11.7 to 22.7)	15.4% (10.4 to 21.7)
7 to 9 months ago	5.9% (1.2 to 16.2)	16.7% (6.4 to 32.8)	6.7% (1.4 to 18.3)	9.9% (6.1 to 15.0)	4.6% (2.0 to 8.8)
10 to 12 months ago	2.0% (0.0 to 10.5)	11.1% (3.1 to 26.1)	13.3% (5.1 to 26.8)	10.9% (6.9 to 16.2)	12.6% (8.0 to 18.4)
>12 months ago	78.4% (64.7 to 88.7)	41.7% (25.5 to 59.2)	17.8% (8.0 to 32.1)	34.4% (27.7 to 41.6)	37.7% (30.5 to 45.3)
**Location of injury**	*n* = 50		*n* = 44	*n* = 191	
**Practice**	**92.0% (80.8 to 87.8)**	**97.2% (85.5 to 99.9)**	**97.7% (88.0 to 99.9)**	**100.0%**	**96.0% (91.9 to 98.4)**
Bathroom		2.8% (0.1 to 14.5)		0.5% (0.0 to 2.9)	
Car park	6.0% (1.3 to 16.6)	5.6% (0.7 to 18.7)	2.3% (0.1 to 12.1)	1.6% (0.3 to 4.5)	1.1% (0.1 to 4.1)
Consult/examination room	4.0% (0.5 to 13.7)	22.2% (10.1 to 39.2)	18.2% (8.2 to 32.7)	15.2% (10.4 to 21.1)	46.9% (39.3 to 54.5)
Corridor		8.3% (1.8 to 22.5)		1.6% (0.3 to 4.5)	1.1% (0.1 to 4.1)
Dispensary		2.8% (0.1 to 14.5)	2.3% (0.1 to 12.1)	1.6% (0.3 to 4.5)	1.1% (0.1 to 4.1)
Isolation				0.5% (0.0 to 2.9)	
Kitchen	2.0% (0.1 to 10.7)	8.3% (1.8 to 22.5)			0.6% (0.0 to 3.1)
Laundry	2.0% (0.1 to 10.7)				
Office	4.0% (0.5 to 13.7)	5.6% (0.7 to 18.7)			
Outside	12.0% (4.5 to 24.3)	2.8% (0.1 to 14.5)	2.3% (0.1 to 12.1)	0.5% (0.0 to 2.9)	
Practice flat				0.5% (0.0 to 2.9)	
Prep room	32.0% (19.5 to 46.7)	19.4% (8.2 to 36.0)	45.5% (30.4 to 61.2)	51.3% (44.0 to 58.6)	30.3% (23.6 to 37.7)
Radiography			6.8% (1.4 to 18.7)	0.5% (0.0 to 2.9)	1.1% (0.1 to 4.1)
Reception	10.0% (3.3 to 21.8)	16.7% (6.4 to 32.8)	2.3% (0.1 to 12.1)	1.6% (0.3 to 4.5)	2.3% (0.6 to 5.7)
Staff room					0.6% (0.0 to 3.1)
Theatre	2.0% (0.1 to 10.7)		2.3% (0.1 to 12.1)	3.7% (1.5 to 7.4)	3.4% (1.3 to 7.3)
Utility room	4.0% (0.5 to 13.7)		2.3% (0.1 to 12.1)		
Ward/kennels	14.0% (5.8 to 26.7)		13.6% (5.2 to 27.4)	18.8% (13.6 to 25.1)	6.3% (3.2 to 11.0)
Unknown		2.8% (0.1 to 14.5)		0.5% (0.0 to 2.9)	
**Non‐practice location**	**8.0% (2.2 to 19.2)**	**2.8% (0.1 to 14.5)**	**2.3% (0.1 to 12.1)**	**0%**	**4.0% (1.6 to 8.1)**
Animal shelter					1.1% (0.1 to 4.1)
Educational institutes	2.0% (0.1 to 10.7)	2.8% (0.1 to 14.5)			0.6% (0.0 to 3.1)
Farm					0.6% (0.0 to 3.1)
Home visit			2.3% (0.1 to 12.1)		
Mobile clinic					0.6% (0.0 to 3.1)
Stable					0.6% (0.0 to 3.1)
Zoo	2.0% (0.1 to 10.7)				0.6% (0.0 to 3.1)
Unknown	4.0% (0.5 to 13.7)				
**Animal involvement in injury**	*n* = 48			*n* = 189	*n* = 173
None	47.9% (33.3 to 62.8)	58.3% (40.8 to 74.5)	22.7% (11.5 to 37.8)	21.2% (15.6 to 27.7)	18.5% (13.0 to 24.8)
Cat	27.1% (15.3 to 41.9)	19.4% (8.2 to 36.0)	43.2% (28.4 to 59.0)	50.3% (42.9 to 57.6)	43.9% (36.4 to 51.7)
Cow					1.2% (0.1 to 4.1)
Dog	22.9% (12.0 to 37.3)	22.2% (10.1 to 39.2)	27.3% (15.0 to 42.8)	25.9% (19.8 to 32.8)	33.5% (26.5 to 41.1)
Ferret	2.1% (0.1 to 11.1)			0.5% (0.0 to 2.9)	0.6% (0.0 to 3.2)
Gerbil			2.3% (0.1 to 12.0)	0.5% (0.0 to 2.9)	0.6% (0.0 to 3.2)
Hamster				0.5% (0.0 to 2.9)	0.6% (0.0 to 3.2)
Horse					0.6% (0.0 to 3.2)
Lemur					0.6% (0.0 to 3.2)
Rabbit				0.5% (0.0 to 2.9)	
Raptor			2.3% (0.1 to 12.0)		
Squirrel			2.3% (0.1 to 12.0)	0.5% (0.0 to 2.9)	
**Mechanism of injury**	*n* = 48	*n* = 35	*n* = 41	*n* = 182	*n* = 171
**Animal injured person** ^†^	**50.0% (35.2 to 64.8)**	**42.9% (26.3 to 60.7)**	**68.3% (51.9 to 81.9)**	**74.2% (67.2 to 80.4)**	**73.0% (65.8 to 79.6)**
Administering oral medications				0.5% (0.0 to 3.0)	1.8% (0.4 to 5.0)
Anaesthesia/induction/recovery			2.4% (0.1 to 12.9)	8.2% (4.7 to 13.2)	4.1% (1.7 to 8.3)
Blood sampling			2.4% (0.1 to 12.9)	1.1% (0.1 to 3.9)	2.3% (0.6 to 5.9)
Clipping nails		2.9% (0.1 to 14.9)	2.4% (0.1 to 12.9)	1.1% (0.1 to 3.9)	1.8% (0.4 to 5.0)
Clinical examination	8.3% (2.3 to 20.0)	5.7% (0.7 to 19.2)	2.4% (0.1 to 12.9)	4.9% (2.3 to 9.2)	29.2% (22.6 to 36.7)
Clinical procedure (unknown sedation status)					0.6% (0.0 to 3.2)
Clinical procedure (with sedation)	4.2% (0.5 to 14.3)		2.4% (0.1 to 12.9)		1.8% (0.4 to 5.0)
General petting	2.1% (0.1 to 11.1)	2.9% (0.1 to 14.9)		1.6% (0.3 to 4.7)	1.2% (0.1 to 4.2)
Handling	4.2% (0.5 to 14.3)	2.9% (0.1 to 14.9)	7.3% (1.5 to 19.9)	3.8% (1.6 to 7.8)	5.8% (2.8 to 10.5)
Injecting IV or IV cannula placement	2.1% (0.1 to 11.1)		4.9% (0.6 to 16.5)	4.4% (1.9 to 8.5)	2.3% (0.6 to 5.9)
Injecting subcutaneously or intramuscularly			2.4% (0.1 to 12.9)		4.7% (2.0 to 9.0)
Injecting (unknown route)					1.8% (0.4 to 5.0)
Kennel cough vaccine administration					1.2% (0.1 to 4.2)
Muzzling			2.4% (0.1 to 12.9)	2.2% (0.6 to 5.5)	
“Out of nowhere” attack	2.1% (0.1 to 11.1)	5.7% (0.7 to 19.2)		1.1% (0.1 to 3.9)	2.3% (0.6 to 5.9)
Radiography					0.6% (0.0 to 3.2)
Restraint	12.5% (4.7 to 25.3)	22.9% (10.4 to 40.1)	24.4% (12.4 to 40.3)	38.5% (31.4 to 46.0)	7.6% (4.1 to 12.7)
Retrieving‐Inserting from patient accommodation	4.2% (0.5 to 14.3)		7.3% (1.5 to 19.9)	2.2% (0.6 to 5.5)	1.2% (0.1 to 4.2)
Retrieving or inserting animal from pet carrier	4.2% (0.5 to 14.3)		2.4% (0.1 to 12.9)	1.1% (0.1 to 3.9)	2.9% (1.0 to 6.7)
Other	2.1% (0.1 to 11.1)		2.4% (0.1 to 12.9)	1.6% (0.3 to 4.7)	
Unknown			2.4% (0.1 to 12.9)	1.6% (0.3 to 4.7)	
**Non‐animal injury**	**50.0% (35.2 to 64.8)**	**57.1% (39.4 to 73.7)**	**31.7% (18.1 to 48.1)**	**25.8% (19.6 to 32.8)**	**26.9% (20.4 to 34.2)**
Contact with harmful substance	2.1% (0.1 to 11.1)			2.2% (0.6 to 5.5)	1.2% (0.1 to 4.2)
Contact with machinery					0.6% (0.0 to 3.2)
Cut by surgical sharp				0.5% (0.0 to 3.0)	1.8% (0.4 to 5.0)
Cut by non‐surgical sharp	2.1% (0.1 to 11.1)	2.9% (0.1 to 14.9)	2.4% (0.1 to 12.9)	1.6% (0.3 to 4.7)	1.8% (0.4 to 5.0)
Exposure to fire				0.5% (0.0 to 3.0)	
Exposure to heat/steam		8.6% (1.8 to 23.1)			0.6% (0.0 to 3.2)
Manual handling	2.1% (0.1 to 11.1)	5.7% (0.7 to 19.2)	2.4% (0.1 to 12.9)	1.6% (0.3 to 4.7)	
Needlestick	2.1% (0.1 to 11.1)	2.9% (0.1 to 14.9)	14.6% (5.6 to 29.2)	6.6% (3.5 to 11.2)	15.8% (10.7 to 22.1)
Musculoskeletal	2.1% (0.1 to 11.1)				
Slips/Trips	18.8% (9.0 to 32.6)	20.0% (8.4 to 36.9)		3.3% (1.2 to 7.0)	1.2% (0.1 to 4.2)
Struck by equipment/furniture	20.8% (10.5 to 35.0)	17.1% (6.6 to 33.7)	9.8% (2.7 to 23.1)	9.3% (5.5 to 14.5)	3.5% (1.3 to 7.5)
Unknown			2.4% (0.1 to 12.9)		0.6% (0.0 to 3.2)

^†^ Will be lower than animal involvement in injury. For example, sharps are not classified as having animal involvement, but if they occurred during surgery, an individual may say an animal was involved

### Mechanism of injury

Administrative staff were injured in highly varied ways, with no clear discernible patterns (Table 3). The top mechanisms of injury were “struck by equipment or furniture”, “slips/trips” and “restraint of an animal”. Animals were not involved in 57.1% of receptionists’ injuries; however, the top injury mechanism was “restraint of an animal”, followed by “slips/trips” and “struck by equipment/furniture”. When animals were involved with injuries to these job roles, they were almost exclusively cats and dogs. The majority of ACAs’ injuries involved animals; cats were the most prevalent animal involved, followed by dogs. Around a quarter of all injuries involved the restraint of an animal; these all involved the restraint for a veterinarian to carry out a procedure. There was no obvious trend for procedure type; however, cats were involved in 80% of these incidences. The only other injury mechanism with a prevalence higher than 10% was needlestick injuries.

The majority of injuries to VNs were animal‐related, with around half involving cats and dogs being the next most prevalent animals involved. Injuries mainly occurred when the VN was restraining an animal; in 83.0% of recent and 82.1% of severe injuries, these were cats, with the remainder being dogs. The top three reasons for cats being restrained were for blood samples, injections and clinical examinations. The next most prevalent injury mechanism was during an animal’s “anaesthesia/induction/recovery”; in 73.3% of recent and 76.5% of severe injuries, this led to a bite, and in 46.7% and 47.0% of cases, it was stated that the “animal suddenly woke up”. Similarly, the majority of injuries to veterinarians were caused by animals, with cats being more prevalent in the most recent injuries and dogs being more prevalent in the most severe injuries. Clinical examinations were the most common activity occurring at the point of injury; 51.8% recent and 40.9% severe were examinations of the head, anal examinations (11.1% recent, 18.2% severe), chest examinations (11.1% recent, 9.1% severe) and abdominal examinations (11.1% recent, 4.5% severe). Needlesticks were the next most prevalent injury mechanism, representing almost 1 in 6 of all recent injuries to veterinarians.

Needlesticks represented a large burden of injury across all roles (Table 4). Many needlestick injuries occurred whilst performing an injection; however, a concerning number occurred through bad practice (i.e. recapping of needles, passing the device or sharps being in regular waste). Additionally, when active ingredients were mentioned, some included immunosuppressants and sedatives.

**Table 4:** Needlestick injury prevalence and mechanisms within companion animal practices

	Recent injuries (*n* = 47)	Severe injuries (*n* = 22)
**Overall prevalence**	9.6% of all injuries	4.8% of all injuries
**Mechanism of needlestick**		
Injecting	23.4%	31.8%
Recapping	21.3%	31.8%
Fine needle aspirates	8.5%	9.1%
Blood sample	8.5%	
Drawing up	8.5%	
Intravenous cannula placement	6.4%	4.5%
Handling/passing device	6.4%	
Handling waste (sharps bin/bag)	4.3%	
Knocked over by animal	2.1%	4.5%
Unknown	10.6%	18.2%
**Active ingredients mentioned:**	Cyclosporine Methadone Pentobarbital Propofol Vaccines (Nobivac Tricat Trio, Nobivac L4)	Meloxicam Methadone Pentobarbital Propofol

### Context of animal‐related injuries

In 21.4% of recent, and 18.2% of severe, animal‐related injuries, the injured person described the animal “attacking out of nowhere” or that it “suddenly bit” or that there were no behavioural warning signs.

*“Was trying to look at a wound and dog went for my arm with no warning.”* – VN, dog bite to arm, received first aid

In 9.3% of recent, and 9.5% of severe, animal‐related injuries the injured person described that the animal was fearful/anxious preceding the injury.

*“Dog was a bit nervous but the owner said she liked humans, just dogs she didn’t. As I just had palpated her abdomen and was raising myself up she launched at me and bit my face.”* – VS, dog bite to face, received first aid

When another person was present at the point of injury, 2.7% of recent and 5.3% severe injury circumstances, the injured person blamed the other individual for the injury occurring.

*“I was holding a German Shepherd Dog for a painful intramuscular injection, explained to Vet I didnt have a good hold on him but Vet went ahead and injected, dog then bit me on forearm”* *–* VN, dog bite to arm, received first aid

“*Vet asked me to pick up a cat that did not wish to be picked up… I now do not listen to vets when they say a cat is nice if they ask for help during a consult”* – VN, cat bite to hand, attendance at minor injuries unit

Muzzling of an animal was only mentioned in 2.2% of recent animal‐related injuries; however, this rose to 5.7% when related to severe injuries. The common mechanism for muzzle related injuries were, in order of frequency; the dog bit through the muzzle, the dog bit when the muzzle was being putting on, the dog removed the muzzle and that the dog was in post anaesthetic recovery when it was being muzzled.

*“Bitten by an aggressive dog when we were trying to euthanise him. He had a muzzle on and had been given heavy sedatives but he completely freaked out, got the muzzle off and attached himself to my arm”* – VN, dog bite to arm, visited hospital emergency department

In a few circumstances the owner of the dog was asked to muzzle the dog, which they refused, and subsequently, veterinary staff were injured.

*“Vet wanted bloods done in consult in front of owner, wouldn’t allow muzzle to be put on, dog chewed on my finger”* – VS, dog bite to arm, attended primary care physician

### Injury consequences and behavioural response to injury

Overall, 1.0% (95% CI 0.3 to 2.4) of companion animal practice staff’s most recent work‐related injury was reportable to the HSE through RIDDOR as a “specified injury”. This did not vary between roles: administrative 4.2% (95% CI 0.5 to 14.3), receptionist 2.9% (95% CI 0.1 to 14.9), ACA’s 2.3% (95% CI 0.1 to 12.3) and VS 0.5% (95% CI 0.0 to 3.0); VNs had no reportable injuries. However, when looking at their most severe injuries, 4.3% (95% CI 2.7 to 6.6) were RIDDOR reportable as a “specified injury”. This did not vary between roles: administrative 6.7% (95% CI 1.4 to 18.3), receptionist 3.2% (95% CI 0.1 to 16.7), ACA 2.6% (95% CI 0.1 to 13.5), VN 3.5% (95% CI 1.3 to 7.4) and VS 5.3% (95% CI 2.4 to 9.8).

As most animal‐related injuries occurred in veterinarians and VNs and were related to cats and dogs, we focused on these groups to understand the anatomical distribution of injuries. Top cat‐related injuries to VNs were bites to hands (recent: 48.4%, severe: 56.6%), scratches to hands (recent: 19.8%, severe: 10.8%), scratches to arms (recent: 13.2%, severe: 9.6%) and bites to arms (recent: 12.1%, severe: 10.8%). Whilst those to vets were bites to hands (recent: 62.9%, severe: 63.1%), scratches to hands (recent: 23.5%, severe: 8.7%), bites to arms (recent: 11.8%, severe: 10.9%) and scratches to arms (recent: 11.8%, severe: 8.7%) (Table S2).

Top dog‐related injuries to VNs involved bites to hands (recent: 38.3%, severe: 30.0%), bites to arms (recent: 26.8%, severe: 26.0%), scratches to arms (recent: 17.1%, severe: 2.0%), ergonomic injuries (recent: 4.9%, severe: 14.0%) and bites to heads (recent: 4.9%, severe: 6.0%). Whilst those to vets were bites to hands (recent: 47.0%, severe: 51.4%), bites to arms (recent: 20.4%, severe: 17.1%) and bites to the head (recent: 8.2%, severe: 12.8%). Two per cent of severe VN dog‐related injuries resulted in concussion, and a further 2% resulted in fracture. Four per cent of veterinarians’ most recent dog‐related injuries resulted in fractures; this was similar for the most severe injuries (4.2%). Whilst 5.7% of severe injuries were degloving. On exploration of the events preceding bites to the head, they all occurred whilst in the waiting or consult room and were associated with either the clinical examination of the head or the dog being passed in the owner’s arms to the VS or VN. Owners were always present for this type of injury.

Most staff were injured with someone else present (Tables 5 and S3). For all roles, excluding veterinarians, the main individuals present were either VNs or VS. In contrast, for veterinarians, the main individuals present at injury were an animal’s owner or VN. The minority of injuries did not receive medical attention. The main form of medical attention for all roles was self‐administered first aid. Dependent on role, between 16.3% (VN) and 23.0% (receptionists) of staff attended or were admitted to hospital for their most recent injury. Considering their most severe injuries, this increased to between 25.8% (receptionists) and 36.9% (VN).

**Table 5:** Consequences of recent work‐related injuries in companion animal veterinary practices

	Administrative staff (*n* = 48) (95% CI)	Reception staff (*n* = 35) (95% CI)	Animal care assistants (*n* = 40) (95% CI)	Veterinary nurses (*n* = 179) (95% CI)	Veterinary surgeons (*n* = 171) (95% CI)
**Was anyone else present at point of injury?** ^†^					
No	29.6% (17.0 to 44.1)	23.5% (10.8 to 41.2)	15.0% (5.7 to 29.8)	19.6% (14.0 to 26.1)	8.2% (4.5 to 13.4)
Admin	2.1% (0.1 to 11.1)	2.9% (0.1 to 14.9)		1.1% (0.1 to 4.0)	
Animal care assistant	4.2% (0.5 to 14.3)		5.0% (0.6 to 16.9)	4.5% (1.9 to 8.6)	1.2% (0.1 to 4.2)
Owner or client	10.4% (3.5 to 22.7)	11.4% (3.2 to 26.7)	10.0% (2.8 to 23.7)	5.0% (2.3 to 9.3)	38.0% (30.7 to 45.7)
Receptionist	6.3% (1.3 to 17.2)	8.8% (1.9 to 23.7)	2.5% (0.1 to 13.2)	2.2% (0.6 to 5.6)	1.2% (0.1 to 4.2)
Student veterinary nurse			5.0% (0.6 to 16.9)	4.5% (1.9 to 8.6)	2.3% (0.6 to 5.9)
Veterinary nurse	22.9% (12.0 to 37.3)	29.4% (15.1 to 47.5)	30.0% (16.6 to 46.5)	26.8% (20.5 to 33.9)	44.4% (36.9 to 52.2)
Veterinary student	4.2% (0.5 to 14.3)	2.9% (0.1 to 14.9)		0.6% (0.0 to 3.1)	
Veterinary surgeon	20.8% (10.5 to 35.0)	14.3% (4.8 to 30.3)	47.5% (31.5 to 63.9)	44.7% (37.3 to 52.3)	8.8% (5.0 to 14.1)
Other					
**Medical treatment**				n=178	
None	8.3% (2.3 to 20.0)	14.3% (4.8 to 30.3)	5.0% (0.6 to 16.9)	7.3% (3.9 to 12.2)	21.6% (15.7 to 28.6)
Self‐administered first aid	58.3% (43.2 to 72.4)	34.3% (19.1 to 52.2)	52.5% (36.1 to 68.5)	55.6% (48.0 to 63.1)	46.2% (38.6 to 54.0)
First aid received	6.3% (1.3 to 17.2)	22.9% (10.4 to 40.1)	10.0% (2.8 to 23.7)	2.8% (0.9 to 6.4)	2.9% (1.0 to 6.7)
Primary care	4.2% (0.5 to 14.3)	5.7% (0.7 to 19.2)	10.0% (2.8 to 23.7)	17.4% (12.2 to 23.8)	9.9% (5.9 to 15.4)
Minor injury unit	8.3% (2.3 to 20.0)	14.3% (4.8 to 30.3)	17.5% (7.3 to 32.8)	12.4% (7.9 to 18.1)	12.9% (8.2 to 18.8)
Emergency department	10.4% (3.5 to 22.7)	2.9% (0.1 to 14.9)	2.5% (0.1 to 13.2)	2.2% (0.6 to 5.7)	5.3% (2.4 to 9.7)
Hospital admission	4.2% (0.5 to 14.3)	2.9% (0.1 to 14.9)	2.5% (0.1 to 13.2)	1.7% (0.3 to 4.8)	1.2% (0.1 to 4.2)
Hospital outpatients		2.9% (0.1 to 14.9)			
Physiotherapist				0.6 (0.0 to 3.1)	
**Physical recovery time**					
<7 days	66.7% (51.6 to 79.6)	65.7% (47.8 to 80.9)	75.0% (58.8 to 87.3)	65.7% (58.3 to 72.7)	74.9% (67.7 to 81.2)
7 to 14 days	8.3% (2.3 to 20.0)	20.0% (8.4 to 36.9)	17.5% (7.3 to 32.8)	27.0% (20.6 to 34.1)	18.1% (12.7 to 24.7)
15 to 28 days	12.5% (4.7 to 25.3)		5.0% (0.6 to 16.9)	3.9% (1.6 to 7.9)	4.1% (1.7 to 8.3)
>28 days	12.5% (4.7 to 25.3)	14.3% (4.8 to 30.3)	2.5% (0.1 to 13.2)	3.4% (1.2 to 7.2)	2.9% (1.0 to 6.7)
**Time off work**					
None	85.4% (72.2 to 93.9)	88.6% (73.3 to 96.8)	85.0% (70.2 to 94.3)	90.4% (85.2 to 94.3)	91.8% (86.7 to 95.5)
<7 days	4.2% (0.5 to 14.3)		12.5% (4.2 to 26.8)	6.7% (3.5 to 11.5)	7.0% (3.7 to 11.9)
7 to 14 days	2.1% (0.1 to 11.1)	2.9% (0.1 to 14.9)		1.1% (0.1 to 4.0)	0.6% (0.0 to 3.2)
15 to 28 days	2.1% (0.1 to 11.1)	2.9% (0.1 to 14.9)			0.6% (0.0 to 3.2)
>28 days	6.3% (1.3 to 17.2)	5.7% (0.7 to 19.2)	2.5% (0.1 to 13.2)	0.6 (0.0 to 3.1)	
**Was there a practice policy to minimise risk?**					
Yes	41.7% (27.6 to 56.8)	11.4% (3.2 to 26.7)	30.0% (16.6 to 46.5)	15.2% (10.2 to 21.3)	8.8% (5.0 to 14.1)
No/not aware	58.3% (43.2 to 72.4)	88.6% (73.3 to 96.8)	70.0% (53.5 to 83.4)	84.8% (78.7 to 89.8)	91.2% (85.9 to 95.0)
**Did you inform practice management?**	n=47	n=34			
Yes	85.1% (71.7 to 93.8)	76.5% (58.8 to 89.3)	97.5% (86.8 to 99.9)	87.6% (81.9 to 92.1)	69.0% (61.5 to 75.8)
**Did you file an accident/incident report?**					
Yes	61.7% (46.4 to 75.5)	70.6% (52.5 to 84.9)	90.0% (76.3 to 97.2)	74.1% (67.1 to 80.4)	47.4% (39.7 to 55.1)
**Was any subsequent action taken by the practice?**		n=32		n=176	
Yes	23.4% (12.3 to 38.0)	15.6% (5.3 to 32.8)	15.0% (5.7 to 29.8)	18.8% (13.3 to 25.3)	5.3% (2.4 to 9.8)
No	66.0% (50.7 to 79.1)	59.4% (40.7 to 76.3)	65.0% (48.3 to 79.4)	69.9% (62.5 to 76.6)	78.4% (71.4 to 84.3)
Don’t know	8.5% (2.4 to 20.4)	25.0% (11.5 to 43.4)	20.0% (9.1 to 35.7)	18.8% (13.3 to 25.3)	16.4% (11.2 to 22.8)

^†^ These sections won’t always add to 100% as multiple people could be present at an injury

The majority of staff did not experience any mental or emotional effects resultant from the injury (Tables 6 and S4). (Further supportive quotes to this section of results can be found in Supplementary Material S4 when indicated.) When they did, the main effect was “increased anxiety or fear” when in the same situation (Table S4).

*“It was a real shock as I had previously felt fairly confident with dogs and knowing if they were at risk of showing aggression. I am now pretty fearful of German Shepherds in particular! The fact the dog went for my throat was what scared me the most ‐ although the hand injury wasn’t too severe, it could have been severe or impacted my face.”* – VN, dog bite to hand, attended hospital emergency department

However, a range of effects were mentioned including feeling “undervalued by the employer”, being “more cautious around that species of animal”, “embarrassment” and having their “confidence knocked”.

**Table 6:** Reasons for behavioural responses to a recent work‐related injury in companion animal veterinary practices

Themes	Administrators	Receptionists	Animal care assistants	Veterinary nurses	Veterinary surgeons
**Did you experience any** **emotional or mental** **effects resultant of the injury?**	*n* = 47	*n* = 35	*n* = 40	*n* = 178	*n* = 171
None	91.5%	91.4%	90.0%	87.6%	83.6%
Increased anxiety or fear		5.7%	10.0%	6.7%	12.3%
More cautious around the animal				3.9%	4.1%
Won’t work with that species again	2.1%			0.6%	
Confidence knocked				0.6%	0.6%
Embarrassment	2.1%	2.9%		0.6%	1.2%
Annoyance	2.1%			0.6%	
Undervalued by employer	2.1%			1.1%	0.6%
**Why did you** **not take time off** **from work?**	*n* = 40	*n* = 31	*n* = 34	*n* = 161	*n* = 157
Genuinely minor injury not requiring time off work	85.0%	93.5%	97.1%	89.4%	85.4%
Could still function with a reduced workload	10.0%	6.5%		8.1%	8.9%
“Be tough”/“Just get on with it!”				1.9%	1.3%
Don’t want to lose sick leave‐sick pay, etc.	2.5%			0.6%	
Not allowed to by employer					1.3%
Used annual leave	2.5%		2.9%	1.2%	2.5%
Didn’t want to let team down				0.6%	3.2%
**Why did you choose not to report the incident?**	*n* = 18	*n* = 8	*n* = 4	*n* = 45	*n* = 90
Only minor injury, which wasn’t serious enough	22.2%	75.0%	50.0%	64.4%	54.4%
Unaware it needed reporting					3.3%
Didn’t want to make a fuss				2.2%	2.2%
Too much effort as was too busy	16.6%	12.5%		15.6%	16.7%
It would cause tension amongst the team				4.4%	
It’s just one of those things/“par for the course”	5.6%		25.0%	8.9%	8.9%
Not employed at CVS at the time	38.9%	12.5%	25.0%	2.2%	2.2%
I forgot to do it	27.8%			13.3%	12.2%
No reporting system in place	5.6%			2.2%	11.1%
**Have you changed your behaviour around the animal species involved in the injury?**	*n* = 44	*n* = 34	*n* = 39	*n* = 176	*n* = 169
No	59.1%	47.1%	48.7%	47.2%	52.1%
More cautious and less confident	6.8%	2.9%	7.7%	11.4%	9.5%
Avoidance of procedure or animal	2.3%		2.6%	0.6%	2.4%
Do procedure differently	6.8%	14.7%	15.4%	14.8%	10.1%
Improve handling/restraint	6.8%	2.9%	15.4%	10.8%	9.5%
Would now use personal protective equipment or a muzzle	11.4%	2.9%	7.7%	9.1%	11.2%
Denial that did anything wrong	2.3%			3.4%	3.0%
Improved awareness of surroundings	9.1%	14.7%	5.1%	5.1%	4.7%

More than a quarter of all roles felt they needed more than 7 days to physically recover from their most recent injury (Table 5). This increased when the injury was more severe, most notably in VN and VS in which 64.2% and 57.3%, respectively, needed more than 7 days. However, there was discordance between time to recover and time taken off work. Overall, less than 14% of injured persons took more than 7 days of work. This discordance was most noted in severe injuries to veterinarians, in which <5% took more than 7 days off work. This was despite almost 10% of the injuries involving fractures or degloving’s. The main reason the injured party did not want to take time off was that the injury was genuinely minor, and it did not require time off work (Table 6). Overall, this reason accounted for more than 85% of the responses. Other reasons included that they “did not want to let the team down”:

*“I could still drive and was physically able to work, and didn’t want my colleagues to have to do my work for me.”* – VS, dog bite to face, attended minor injuries unit

In some circumstances, they felt that they could still work, but with a reduced workload (Table S4), a few gave the impression that they needed to “be tough” and “get on with it” (Table S4):

*“I can fight through it, it is imperative that I work”* – VS, manual handling injury, attendance at hospital emergency department

In a minority of circumstances, the injured persons manager would not let them take time off work:

*“I asked to but was told my practice would give me light duties. They didn’t.”* – VS, chronic shoulder tendonitis, attendance at primary care physician

In all roles, except veterinarians, the majority of respondents reported their injury via an official accident book or injury reporting system. Most veterinarians did not report their injuries. The main three reasons given were; It was “too much effort” or they were “too busy” to fill in the report (Table S4);

*“I did not have time for paperwork and I could manage it myself I didn’t need people checking up on me”* – VS, cat bite to hand, received first aid

Some veterinarians saw that their injury was inevitable and just an everyday hazard not worthy of reporting and some just forgot to report it (Table S4);

*“Part of the job. Plus far too many questions and not enough time to complete, then get chased for more info I don’t have time to answer”* *–* VS, dog bite to hand, attendance at hospital emergency department

*“It was less of conscious decision and more due to the fact that it was a busy day with more pressing matters to attend to and by the next day reporting the incident was forgotten about.”* – VS, dog bite to hand, received first aid

In a minority of cases, respondents did not want to be perceived differently by their colleagues;

*“I was much less confident about reporting and didn’t want to make a fuss or seem high maintenance therefore I got on with it”* – VS, cat bite to hand, no treatment

Around half of respondents would change their behaviour if placed in the same scenario, for their most recent injuries, this increased for all roles when the injury was their most severe. This concerningly still meant a high proportion would not change their behaviour, and thus place themselves at risk again. Some individuals would avoid the procedure or animal to totally negate the risk.

*“I try to avoid any contact with fractious cats if I can.”* – VS, cat bite to hand, attendance at hospital emergency department

*“I’m no longer allowed to handle squirrels”* – ACA, squirrel bites to hand and arms, attendance at hospital emergency department

The change in behaviour was split between four key themes. Firstly, that the respondent was “more cautious, and less confident” in the same situation (Table S4);

“*I’ll just be more careful when resheathing needles*” – VN, needlestick to hand, received first aid

Some respondents would carry out the procedure differently (Table S4);

*“I read a lot and learnt a lot about dog behaviour so I am much more aware of body language and I would never put myself in a similar position again”* – VS, dog bite to hand, received first aid

Many would improve their restraint techniques (Table S4);

“*Now will try to towel wrap and also dispense ‘chill protocol’ prior to appointment*.” – VN, cat scratch to arm, received first aid

Finally, many would now start muzzling dogs on a more regular basis prior to consultations, examinations or procedures (Table S4);

*“Muzzle more often and insist on them despite owner protests of ‘he doesn’t bite’, as this is followed by ‘he hasn’t done that before’”* – VS, dog bite to head, attendance at minor injury unit

Most respondents were not aware of, or did not think there was, any practice policy in place that could have mitigated their injury. Policies when mentioned included; cat and dog handling guidelines, feral cat and wildlife handling guidelines, and general personal protective equipment (PPE) guidelines. Similarly, when asked whether the practice, where they were employed at the time, took any action after the incident described, the majority said that no action was taken, or they were not aware of any action. When actions were described they included; a review of current risk assessments, a review of the incident, workload and rota adjusted to accommodate injured person, new practice muzzle policy, new sedated dogs protocol, review of chemical dilutions protocols, review of cleaning protocol, provision of animal handling training for all staff and new PPE was provided.

## DISCUSSION

This is one of the largest studies to explore work‐related injuries within companion animal veterinary practices. Roles directly involving animal interactions had a higher annual prevalence of injury (Mills, 2015), a higher frequency of injuries (Johnson & Fritschi, 2024; Tulloch et al., 2025) and were predominantly injured by animals (Epp & Waldner, 2012; Fritschi et al., 2006; Gabel & Gerberich, 2002; Johnson & Fritschi, 2024; Nienhaus et al., 2005; Tulloch et al., 2023). Thus, this shows that animals are the main driver of injuries for veterinarians, nurses and ACAs (Fowler et al., 2016). This is emphasised by our model where job role was the main significant variable associated with receiving an injury. This model showed that hours worked was an important variable, with roles undertaking <20 hours or >40 hours of work a week at a generally lower risk of obtaining injuries compared to roles working 21 to 40 hours a week; however, this relationship was non‐linear and challenging to interpret. Further research is needed to understand this relationship, though it could be linked to differences in experience, animal exposure or task allocation.

The importance of a positive safety culture in reducing workplace injuries has been widely acknowledged (Guldenmund, 2000). A positive safety culture comprises four components: a reporting culture, a just culture, a flexible culture and a learning culture (Reason, 1997). A “reporting culture” is a workplace environment where staff feel safe and encouraged to report injuries, near misses and hazards without fear of reprisals. A “just culture” balances accountability and learning, distinguishing between acceptable errors and unacceptable behaviours. This aids in generating credibility and confidence in the organisation. A “flexible culture” is one where an organisation adapts quickly to changing circumstances and crises, ensuring safety is maintained under varying conditions. A “learning culture” is one where the employer and employee want to both improve safety and are willing, and allowed, to reflect on their own behaviour and that of others. It shows a commitment to analysing incidents, sharing lessons and continuously improving safety practices (European Agency for Safety and Health at Work, 2023). Throughout the discussion, we will use this framework to highlight where we identified behaviours supporting or opposing a positive safety culture.

Around half of injuries to receptionists and administrators involved animals, primarily restraint of the animal. This highlighted the multi‐faceted nature of these roles and that within a companion animal clinical setting, duties may not be fully clerical in nature. It is important that those in these roles, without prior veterinary experience, have been trained appropriately in how to handle and restrain animals. The remainder of injuries matched similar national workplace injury trends, with slips, trips and strikes by equipment or furniture being the most common cause of injury (Jabbour et al., 2015; Lundstrom et al., 2023). Standard injury prevention guidance should be followed to minimise the risk of these injuries (Health & Safety Executive, 2012).

The practice location of injuries (prep room, consulting room, ward) was reflective of where ACAs, VNs and VS spend much of their time. This indicates that any interventions developed to minimise work‐related injuries for clinical staff should be focused in these areas. It is unsurprising that most injuries were animal‐related, with the main species involved being cats and dogs (Johnson & Fritschi, 2024; Tulloch et al., 2023). Restraint of cats appeared to be a particular issue, especially when they were being held by VNs and ACAs for blood samples, injections and clinical examinations. Cat‐friendly veterinary interaction guidelines do exist (Rodan et al., 2022) and an estimated 37% of UK practices (based on survey responses) are designated cat‐friendly clinics (Feilberg et al., 2021). Restraining cats can be challenging and unpredictable; yet, these results indicated that potentially this guidance and training may not be working or adhered to in these situations and that more training is needed across the profession to improve cat handling and restraint. During these restraint procedures, it was notable that some injured persons blamed the other person involved in the restraint or veterinary procedure for the injury. This often led to a lack of trust in the “blamed” individual. This potentially evidenced team miscommunication and coordination and differing expectations of their colleagues’ anticipated movements during the restraint process. Since some respondents attributed their injury to a colleague’s actions, it is possible that they do not see the need to learn or adapt their subsequent behaviour as it was out of their own personal control. A blame culture can harm team dynamics and lead to workplace tension. A shift towards a “just” and “learning” culture could minimise injuries and improve communication. This would involve using a team‐based approach where anyone involved in restraint had clearly defined roles and responsibilities. Inclusive reflection post‐injury or near‐miss incident would facilitate learning opportunities to improve restraint and could enable discussions about what could be done differently rather than attributing blame. Practices should foster a work environment where safety concerns can be raised openly and constructively without the potential fear of punishment, thus instilling a “just culture” in the workplace.

VNs and VS were frequently injured when animals were recovering from anaesthesia, with the animal “suddenly waking up.” This is similar to an audit of UK veterinary schools where they found that 64% of all head injuries caused by dogs were associated with dogs anaesthetic recovery or extubation (Tulloch et al., 2023). These results raise questions about the recovery environment, including anaesthetic depth, quality of pain relief, recognition of signs of disorientation, environmental calmness and monitoring levels. Anaesthesia and monitoring guidelines do exist (Grubb et al., 2020); however, they primarily focus on patient safety and do not discuss the potential of injury to veterinary professionals nor how they can be reduced. Further research is needed to understand what the most appropriate preventative measures are, and how they can balance patient safety and occupational safety.

Clinical examinations were the most common activity occurring at the point of injury for VS. Examinations to the head represented around half of these. These are likely due to defensive reactions, pain sensitivity and restricted vision of the animal. Proper restraint, gentle handling and a calm approach could help minimise risk. Understanding animal behaviour is key to reducing animal‐related injuries during a consultation. One in five recent injuries was described as “out of nowhere” attacks, where the IP reported no behavioural warning signs. In other incidents fear or anxiousness of the animal was seen, but no adjustment behaviour by the IP was subsequently described to reduce the likelihood of a negative physical interaction. Dogs and cats display complex and subtle emotional signals that may not be overtly visible during a consultation or be difficult to interpret (Demirbas et al., 2016; Ellis, 2018; Walsh et al., 2024). If these signs were missed, then it could appear that the animal attacked “out of nowhere.” To reduce injuries from animals that are in fear or pain or are aggressive, a consultation environment needs to be created that enables low‐stress handling techniques and reduces miscommunication between humans and animals. This could be facilitated through further animal behaviour training of those with roles who interact with animals, alongside interventions that could minimise the risk of injury, such as using muzzles. If muzzling or other PPE restraint mechanisms are used, then complementary education of owners is required so that an appreciation for the muzzles’ need is acquired. Practice policy should be developed that supports VS and VNs who request a muzzle for a patient, but where an owner declines. Additionally, muzzles should be used more generally with appropriate sizes and fits for all types of dogs. As seen in our data, dogs can bite through a muzzle if it is incorrectly fitted.

The high prevalence of needlesticks is a concern; almost 10% of all injuries and 1 in 6 of VS injuries. This is lower than reported in Australian VSs (57%) and VNs (75%) (Johnson & Fritschi, 2024). This could be explained by the safety culture in the UK veterinary profession, also seen in Canada (Weese & Jack, 2008). The veterinary professionals in our study have previously described needlestick injuries as a common incident not worthy of reporting (Furtado et al., 2024). They also described needlesticks not fulfilling their criteria of an injury, namely that they were common, minor, self‐attributed and not causing lasting pain. As such, it is likely that these prevalence figures represent a large underreporting of the true prevalence. In our results, a third of these injuries were through not following recommended guidance (i.e. recapping of needles, passing the device or sharps being placed in regular waste). These injuries pose a risk to IPs through exposure to zoonotic infections (i.e. *Brucella canis*) and potential exposure to bloodborne pathogens (i.e. hepatitis) (Robertson et al., 2016). More common is that they can lead to injection site reactions, abscess formation or secondary ascending infections if bacterial pathogens enter the wound (Robertson et al., 2016). Accidental injections of veterinary drugs could cause serious health effects (including allergic reactions or systemic toxicity), and many of the drugs discussed in our results were immunosuppressants and sedatives. Needlestick injuries, whilst common in veterinary practice, should always be taken seriously to prevent long‐term health risks. More research is needed to understand the veterinary profession’s attitudes towards needlestick injuries, to best develop appropriate workplace safety improvements.

Only a small proportion of injuries experienced were a RIDDOR reportable injury, in contrast to equine and production animal vets where 11.6% and 13.3% of their respective injuries were reportable (Tulloch et al., 2025). Cat‐related injuries were predominately bites to hands and scratches to hands and arms, consistent with previous literature (Colmers‐Gray et al., 2023; Fritschi et al., 2006; Kheiran et al., 2019; Tulloch et al., 2023). Twenty‐eight per cent of cat bite injuries led to attendance or admission at a hospital; this is much higher than the 5% reported at UK veterinary schools (Tulloch et al., 2023). Current recommendations are that most cat bites should receive antibiotic prophylaxis due to their high risk of infection (Colmers‐Gray et al., 2023). These figures represent an improvement from previous literature, but further education of the veterinary community is needed to encourage rapid assessment of cat bite injuries at emergency departments. Dog‐related injuries were mainly bites to hands and arms, as per the literature (Colmers‐Gray et al., 2023; Fritschi et al., 2006; Tulloch et al., 2021, 2023); however, almost one in ten were bites to heads. Previous research had found that bites to the head were associated with the animal’s anaesthetic recovery (Tulloch et al., 2023), whereas we found that they were associated with clinical examinations of the head and the passing of the dog between the owner and IP. These are both common daily activities in veterinary practice, and investigations need to be performed to assess the best methodology to minimise the injury risk from them.

VNs experienced ergonomic injuries, mostly through lifting heavy patients or equipment. Research from Australia has identified that more than half of VNs suffered from chronic back or neck pain (van Soest & Fritschi, 2004), most likely from poor ergonomic practices. VNs do a lot of heavy lifting of patients, with larger dog breeds being in excess of 50 kg. Lifting heavy patients increases muscular and joint pain, compared to a rigid object, as the dog can unpredictably move or be limp if sedated/unconscious. Practices should encourage team lifting for large dogs and adopt appropriate lifting techniques. The development and utilisation of mechanical lifting aids should be explored to reduce the need for manual lifting and reduce the burden of musculoskeletal injuries on practices.

More than 1 in 10 injuries to VS were lead‐related. These types of injuries can be serious, noted by the fractures to feet and hands seen in our data. The common mechanism for these injuries tends to result from a pull with a subsequent trip or tangle in the lead (Forrester, 2020). These injuries are likely due to a combination of factors including inappropriate lead restraint (i.e. extendable or long leads) and reduced situational awareness. These should represent some of the more preventable injuries within veterinary practices. Solutions could include using team‐based handling for larger or agitated animals and provision of appropriate restraint tools if the dog’s owner does not provide appropriate restraint.

Most individuals did not experience emotional or mental health consequences resultant from the injury. Studies have shown that traumatic workplace injuries are associated with worse mental health trajectories than non‐workplace injuries (Wightman et al., 2025) and that individuals injured at work have worse self‐reported general health and mental health 10 years post‐injury (Dong et al., 2015). Considering that the veterinary profession has a high prevalence of mental health issues (predominantly anxiety and depression) and suicide (Bartram et al., 2009; Platt et al., 2010), it is surprising that most respondents were not impacted by their injuries, especially considering the severity of some of the injuries. Possible reasons for a low prevalence of mental health issues could include denial, being unaware of mental health symptoms or an inability to recognise them, cultural stigma or masking through strong coping mechanisms. Research is needed to understand this issue.

The most common form of medical treatment was self‐administered first aid. However, there was a higher reported attendance at hospital for VNs (16.3%) and VSs (19.4%) than in previous UK literature (5% of cat‐related and 4% of dog‐related injuries to clinical staff at veterinary schools) (Tulloch et al., 2023). For comparison, this was 25.1% for equine veterinarians and 40.0% for production animal veterinarians in general practice (Tulloch et al., 2025). These higher rates of hospital attendance could have multiple potential causes that require further exploration to understand. There could be self‐selection bias, as those who have experienced more severe injuries may have been more inclined to participate in the study. There may be more stringent health and safety standards at the participant’s veterinary practice where the injury occurred, whereby the threshold for hospital attendance is lower than at other organisations. There could be more awareness and reduced stigma around injury, leading to injuries being taken seriously or that these respondents are receiving more serious injuries than the prior literature.

Similar to previous research, there was discordance with the time that an IP took to physically recover and the time taken off work (Tulloch et al., 2025). For most individuals, the injury was minor enough that an IP potentially would not have needed to take time off work. Some felt that they could function with a lightened or adapted workload. In comparison with equine veterinarians, there was much less bravado and dismissive language around severe injuries, and a lower percentage of people feeling guilty about time off work or letting their colleagues down (Tulloch et al., 2025). This potentially indicates that different safety cultures exist between companion animal and large animal vets and that any interventions developed to change these cultures should be tailored to each broad veterinary sector. These results suggest that improvements need to be made in creating a “just” and “flexible” culture.

The veterinary industry as a whole should be aware that working whilst injured increases the risk of reinjury significantly in the first 6 months after injury, but can impact the IP’s health for up to 4 years (Sears et al., 2021). Our results identify a strong culture of presenteeism; this is defined as the act of going to work and working despite health problems (Won et al., 2022). The consequences of which are well described in other industries: individuals will have decreased mental and physical acuity and health, with reduced productivity (Sanderson & Cocker, 2013; Skagen & Collins, 2016). Whilst the practice will have a decline in practice culture, patient care, staff wellbeing, reduced team cohesion and increases in accidents and job turnover (Sanderson & Cocker, 2013). This culture needs to be explored further in the veterinary industry with industry‐specific interventions developed.

Around half of respondents would not alter their behaviour if placed in a similar situation to the one that led to their injury. This proportion is notably higher than that reported in equine and production animal clinical staff (Tulloch et al., 2025). However, respondents who experienced more severe injuries were more likely to report subsequent behavioural changes. When changes were made, these typically involved modifying procedural technique, exercising greater caution and utilising better restraint or PPE. These findings show the need for fostering a stronger culture of shared responsibility and reflective practice within veterinary teams, in essence developing a “learning culture”. Structured debriefs and team‐based safety training may support this shift, ensuring that all staff feel both empowered and accountable for workplace safety.

The awareness and knowledge of practice health and safety protocols and policies were poor, with the majority not knowing of any policy. This, partnered with most VSs not reporting injuries, is indicative of a work safety culture where formal procedures could be poorly communicated or undervalued. This could contribute to underreporting, lack of accountability and missed opportunities for learning and prevention. This is exemplified by the minimisation of reporting by VSs, where they generally felt that reporting took too much effort and that workplace injuries were par for the course. This dismissive tone, often carrying a “badge of honour” attitude, is problematic as it undermines safe working practices and reflects a misunderstanding of the importance of injury reporting for the health and wellbeing of colleagues. This is similar to large animal clinicians who normalise workplace injuries as inevitable (Tulloch et al., 2025). This is suggestive of a poor “reporting culture”.

This work has some limitations. By making all veterinary roles eligible to respond, we limited our ability to explore role‐specific factors and behaviours in depth. Response rates may have improved if we had disseminated role‐specific questionnaires. Future studies on work‐related injuries should focus both on specific roles and the interaction between roles. Whilst a substantial number of individuals completed the survey, some did drop out: 25 VNs, 10 administrators and 7 VS. Anticipating this, we offered an incentive available only upon survey completion. We made clear that we were asking respondents about injuries that may or may not have occurred in CVS practices, their current employers. However, a few participants noted that they did not report the injury in the survey as they were not at CVS at the time. We are also conscious that some individuals may have given responses of what they felt their managers would prefer to hear. However, we believe that this bias was minimal due to the anonymised nature of the survey and that only summary data was shared with CVS. This is evidenced by the openness and honesty of numerous free‐text responses.

This study highlights the high prevalence of work‐related injuries that occur within companion animal practices. We have identified key areas where injuries were common and where research is needed to identify best practices or interventions that can help minimise risk and prevent injuries. These areas include cat handling, general animal restraint, needlestick injuries and animals recovering from anaesthetics. A culture of presenteeism appears to be embedded in these settings, where the personal and institutional impact of injuries is routinely minimised at the potential detriment of all. Since the four components of a positive safety culture are poorly practiced and evidenced, a health and safety cultural shift is needed; one that moves from blame to shared responsibility and reflective learning. Achieving this will require strong organisational leadership and a commitment to fostering an environment where all staff feel they speak openly and take accountability for workplace safety.

### Author contributions

Conceptualisation: J.S.P.T. Funding Acquisition – J.S.P.T., M.W., R.J. Methodology: J.S.P.T., M.W., I.S. Formal analysis: J.S.P.T. Writing original draft – J.S.P.T. Writing review and editing – All authors.

### Conflict of interest

I.S. and R.J. are current employees of CVS UK Ltd.

### Funding information

This work was funded by the CVS Clinical Research Awards (PRA00009, 2022).

## Supporting information


**Data S1:** Supplementary Information.

## Data Availability

The data that support the findings of this study are available from the corresponding author upon reasonable request.
